# Clinically low-risk features do not exclude diffusion-weighted imaging-positive lesions in suspected transient ischemic attack

**DOI:** 10.3389/fneur.2026.1964862

**Published:** 2026-10-14

**Authors:** Leonhard Mann, Kayvan Ern, Simon Stahn, Marcel Hildner, Elke Hattingen, Fee C. Keil

**Affiliations:** 1 Institute of Neuroradiology, University Hospital, Goethe University Frankfurt, Frankfurt am Main, Germany; 2 Department of Radiology, Hospital Nordwest Frankfurt, Frankfurt am Main, Germany; 3 Department of Neurology, University Hospital, Goethe University Frankfurt, Frankfurt am Main, Germany

**Keywords:** clinical risk stratification, diffusion-weighted imaging, DWI, minor stroke, MRI, stroke imaging, TIA, transient ischemic attack

## Abstract

**Introduction:**

Diffusion-weighted imaging (DWI) is essential for distinguishing tissue-negative transient ischemic attack (TIA) from MRI-defined minor stroke. When MRI capacity is limited, clinical features could help estimate DWI lesion yield. While features associated with DWI positivity are well established, it remains uncertain whether clinically low-risk presentations identify patients with a sufficiently low probability of DWI-positive lesions. We investigated clinical features associated with DWI-positive lesions in patients presenting with suspected TIA and assessed whether clinically low-risk constellations reliably excluded DWI-positive lesions.

**Methods:**

We retrospectively analyzed 418 patients presenting with suspected TIA who underwent a standardized MRI protocol within 6 h of admission. The study endpoint was an acute DWI-positive lesion, defined by DWI hyperintensity with corresponding apparent diffusion coefficient reduction. Univariate analyses and exploratory multivariable logistic regression analyses were used to examine clinical, neurological, and cardiovascular features associated with DWI positivity, with particular emphasis on clinically low-risk presentations.

**Results:**

DWI-positive lesions were detected in 118/418 patients (28.2%). Motor symptoms (adjusted odds ratio [aOR] 2.683, *p* < 0.001), NIHSS 1–5 (aOR 1.940, *p* = 0.007), and acute symptom onset (aOR 1.999, *p* = 0.025) were associated with DWI positivity in the clinical-neurological analysis, while cardiovascular risk factors showed the strongest association in the extended analysis (aOR 3.959, *p* < 0.001). Conversely, subacute onset, non-specific accompanying symptoms, NIHSS 0, and dizziness were associated with lower odds of DWI positivity. Importantly, DWI-positive lesions remained present in 13/133 patients (9.8%) with ≥2 and 2/48 (4.2%) with ≥3 low-risk features.

**Discussion:**

Clinically low-risk presentations did not reliably exclude DWI-positive lesions, supporting MRI-based evaluation. Clinical and cardiovascular features may help estimate the likelihood of DWI positivity; whether they can inform MRI prioritization requires prospective validation.

## 1 Introduction

Stroke is a leading cause of mortality, long-term disability, and loss of independence in adulthood (1). As most strokes are ischemic, early recognition and accurate diagnostic classification are essential for timely secondary prevention and risk stratification (2).

In acute stroke imaging, non-contrast cranial computed tomography (CT), often combined with CT angiography and CT perfusion, remains the primary and most widely available diagnostic modality (3, 4). However, CT may miss small acute ischemic lesions, particularly in patients with transient or mild neurological symptoms (4, 5).

This limitation is especially relevant in suspected transient ischemic attack (TIA). According to the modern tissue-based definition, TIA is defined as transient neurological dysfunction caused by focal ischemia of the brain, spinal cord, or retina without evidence of acute infarction (6). Thus, the distinction between tissue-negative TIA and minor stroke depends on the presence or absence of acute ischemic tissue injury rather than symptom duration alone (6).

Magnetic resonance imaging (MRI) with diffusion-weighted imaging (DWI) is central to this distinction. DWI enables sensitive detection of acute ischemic lesions and may identify small infarcts missed on CT (5, 7, 8). In patients with transient or minor neurological symptoms, a DWI-positive lesion supports the diagnosis of MRI-defined minor stroke rather than tissue-negative TIA (6, 7).

Beyond diagnostic classification, MRI may help distinguish ischemic events from non-ischemic mimics and contributes to etiological assessment and early risk stratification (7, 8). However, DWI findings must be interpreted in clinical context. DWI-negative ischemic strokes may occur, particularly in small lesions, posterior circulation events, or very early imaging, and DWI-lesion detection may depend on MRI protocol characteristics and timing (9, 10). Conversely, DWI-positive lesions may be detected even after transient or minor symptoms, with implications for diagnosis, work-up, and secondary prevention.

Clinical classification of transient neurological symptoms therefore remains challenging. Early stroke risk after TIA is highest within the first days, requiring rapid evaluation and timely secondary prevention (11–13). At the same time, non-ischemic differential diagnoses may present with similar symptoms (14, 15). Clinical features such as sudden symptom onset, speech or motor deficits, and vascular risk factors have previously been associated with a cerebrovascular origin (14, 15). However, it remains uncertain whether clinically low-risk constellations identify patients with a sufficiently low probability of DWI-positive lesions to support clinical assessment without MRI.

Therefore, we investigated clinical and cardiovascular features associated with DWI-positive lesions in patients presenting with suspected TIA and assessed whether clinically low-risk constellations reliably excluded DWI-positive lesions.

## 2 Methods

### 2.1 Study design and population

This retrospective, single-center observational study was conducted at the Institute of Neuroradiology of the University Hospital Frankfurt, Germany. The study cohort comprised adult patients clinically assessed by neurologists as having suspected TIA between January 1, 2022, and December 31, 2023, who underwent cranial MRI within 6 h after admission through the institutional TIA diagnostic pathway. Reporting followed the STROBE statement (16).

Patients were included if they were 18 years or older and presented with transient or already regressing neurological symptoms suspicious for TIA, with symptom onset no longer than 14 days before admission. At presentation, symptoms could have completely resolved or already be regressing with residual neurological symptoms. Complete symptom resolution at presentation was not recorded as a separate structured variable in the analytical database.

The analytical database did not retain a screening log documenting the total number of potentially eligible patients, exclusions before database entry with their reasons, or potentially eligible patients who did not undergo MRI within the required timeframe.

Patients were excluded if they had incomplete initial MRI, known intracranial tumors, cerebral metastases, pre-existing inflammatory central nervous system disease, or a documented ischemic stroke within 12 weeks before the index presentation.

### 2.2 Data collection

Clinical and imaging data were collected retrospectively from the electronic medical records, the radiology information system, and the picture archiving and communication system.

For each patient, demographic data, cardiovascular risk factors, cardiac comorbidities, neurological symptoms, National Institutes of Health Stroke Scale (NIHSS), modified Rankin Scale (mRS), use of intravenous thrombolysis, temporal parameters, and imaging findings were systematically recorded in a pseudonymized database. Cardiovascular risk factors were defined as arterial hypertension, diabetes mellitus, dyslipidemia, smoking, obesity, or coronary artery disease. Cardiac comorbidities were defined as atrial fibrillation and patent foramen ovale. For retrospective coding, cardiovascular risk factors were considered present if the structured neurological discharge summary explicitly recorded this category as positive or the discharge diagnoses documented at least one of the predefined vascular risk factors. Cardiac comorbidity was considered present if the corresponding structured category was recorded as positive or atrial fibrillation or patent foramen ovale was documented among the discharge diagnoses.

Time intervals included symptom onset to admission, admission to MRI, and symptom onset to MRI. Symptom onset to MRI interval was calculated from the documented time of symptom onset and the time of MRI acquisition when both time points were available.

Clinical symptoms were documented from NIHSS-related deficits plus dizziness, oculomotor disturbances, and dysphagia, and were coded without weighting. Dizziness was recorded as a binary chart-derived variable based on its documentation in the physician report; the retrospective dataset did not systematically distinguish between specific dizziness phenotypes. Symptom evolution was categorized as acute, subacute, or recurrent. The terms acute and subacute described the mode of symptom development rather than the elapsed time from symptom onset to admission or MRI. Acute onset was defined as sudden onset with immediate neurological dysfunction. Subacute onset was defined as gradual development or worsening of neurological dysfunction. Recurrent symptoms were defined as neurological symptoms that resolved and subsequently recurred.

Non-specific accompanying symptoms, including headache, impaired concentration, nausea, vomiting, and general malaise, were also recorded.

### 2.3 MRI protocol and image assessment

All patients underwent cranial MRI on either a 1.5 T Philips Achieva dStream scanner or a 3-T Siemens Magnetom Skyra fit scanner, using a dedicated head coil. Imaging was performed according to a standardized institutional TIA protocol, including axial and coronal diffusion-weighted imaging with 4-mm slice thickness, 3D time-of-flight MR angiography with 3D reconstructions, T2*-weighted imaging, axial T2-weighted turbo spin echo imaging, and axial FLAIR imaging (Table 1).

**Table 1:** Standardized institutional TIA MRI protocol.

Sequence	Slice thickness (mm)	Acquisition time (min: s)
Axial DWI	4	1:38 min.
Coronal DWI	4	1:39 min.
3D TOF angiography (including 3D reconstructions)	0.9	5:33 min.
Axial T2*-weighted imaging	5	1:00 min.
Axial T2-weighted TSE imaging	5	2:36 min.
Axial T2 FLAIR imaging	5	3:43 min.

Slice thickness and acquisition time of the standardized institutional TIA MRI protocol.

DWI status was extracted from the final approved routine clinical neuroradiology report of the index MRI. All brain MRI examinations were initially interpreted by a physician in training and additionally reviewed by an experienced neuroradiologist. The examinations were also presented and discussed by a further board-certified physician. Clinical readers had access to routine clinical information. Clinical assessment and final neurological diagnosis were performed by experienced neurologists and approved by senior neurologists.

Reported DWI-positive lesions were reviewed by an author for data verification; no blinded study-specific rereading or separate adjudication procedure was performed, and the final approved clinical report served as the reference classification.

### 2.4 Study endpoint

The study endpoint was the presence of an acute DWI-positive ischemic lesion on MRI, defined as hyperintensity on DWI and corresponding apparent diffusion coefficient (ADC) reduction. Patients with transient or mild neurological symptoms and a corresponding acute DWI-positive lesion were classified as having a DWI-positive minor stroke.

### 2.5 Statistical analysis

Statistical analysis was performed using IBM SPSS Statistics, version 31. Continuous variables were analyzed using the independent-samples *t*-test or Mann–Whitney *U* test, depending on distribution. Categorical variables were compared using the Chi^2^ test or Fisher’s exact test. A *p*-value <0.05 was considered statistically significant.

Associations between clinical variables and DWI-positive ischemic lesions were first assessed in univariate analyses, with relative risks calculated for categorical variables. Variables with no DWI-positive events in one subgroup were excluded from multivariable logistic regression. Variable selection was data-driven and based on the results of the univariable analyses together with clinical/pathophysiological relevance. Three exploratory multivariable logistic regression analyses were subsequently performed to assess adjusted associations with DWI positivity:

The first model was a clinical-neurological model including motor symptoms, NIHSS score 1–5, and acute symptom onset to examine their adjusted associations with DWI positivity. The second was an extended clinical-cardiovascular model including motor symptoms, cardiovascular risk factors, cardiac comorbidities, and acute symptom onset; NIHSS score 1–5 was omitted to avoid overlap with motor symptoms, while vascular and cardiac risk factors were additionally assessed. The third was a low-risk model including subacute symptom onset, non-specific accompanying symptoms, NIHSS score 0, and dizziness. It was designed to assess whether combinations of features associated with low DWI positivity could exclude an acute DWI-positive lesion.

Because motor deficits contribute directly to the NIHSS score, motor symptoms and NIHSS score 1–5 were considered partially overlapping. Results from analyses including both variables were therefore interpreted as adjusted associations rather than as evidence of independent predictive effects. Additionally, multicollinearity was assessed using variance inflation factors (VIFs) and tolerance values. Model stability was further assessed in sensitivity analyses by repeating the clinical-neurological model after separately excluding motor symptoms or NIHSS 1–5.

The analyses were exploratory. Low-risk features were selected *post hoc* based on univariable associations, and combined thresholds were evaluated descriptively in the same cohort without internal or external validation or a pre-specified optimization criterion. Sensitivity, specificity, positive predictive value (PPV), negative predictive value (NPV), and DWI-positive proportions were calculated for the evaluated thresholds.

For combined low-risk criteria, exact binomial 95% confidence intervals were calculated.

Multivariable models used complete-case analysis without imputation. Analyses requiring NIHSS data were restricted to patients with a documented NIHSS score. In a sensitivity analysis, symptom-onset-to-MRI time was added to the low-risk model as a continuous covariate, with the effect expressed per 24-h increase.

### 2.6 Artificial intelligence-assisted language editing

ChatGPT (OpenAI) and DeepL were used during manuscript preparation solely for translation, language editing, and improvement of clarity and expression. No scientific content, data analysis, interpretation of results, or conclusions were generated by these tools. The authors reviewed and edited all AI-assisted output and take full responsibility for the final content of the manuscript. These tools were not listed as authors.

## 3 Results

### 3.1 DWI-positive lesions and univariate associations

DWI-positive lesions were identified in 118 of 418 patients, corresponding to 28.2% of the study cohort. Baseline characteristics are summarized in Table 2. The exact symptom onset to MRI interval was available for 279 patients. The median interval was 3.65 h (IQR, 2.07–8.16; range, 0.58–101.33), with no significant difference between DWI-negative and DWI-positive patients (3.66 vs. 3.50 h; *p* = 0.404).

**Table 2:** Study cohort and baseline characteristics.

Domain	Characteristic	Overall cohort	DWI-positive	DWI-negative
Study cohort	Patients, *n*	418	118	300
Demographics	Age >65 years	223/418 (53.3%)	75/118 (63.6%)	148/300 (49.3%)
	Age >80 years	97/418 (23.2%)	30/118 (25.4%)	67/300 (22.3%)
Medical history	Cardiovascular risk factors	289/418 (69.1%)	104/118 (88.1%)	185/300 (61.7%)
	Cardiac comorbidity	76/418 (18.2%)	36/118 (30.5%)	40/300 (13.3%)
Neurological status	NIHSS score = 0	282/405 (69.6%)	64/117 (54.7%)	218/288 (75.7%)
	NIHSS score 1–5	120/405 (29.6%)	51/117 (43.6%)	69/288 (24.0%)
Symptom onset	Acute symptom onset	324/418 (77.5%)	101/118 (85.6%)	223/300 (74.3%)
	Subacute symptom onset	47/418 (11.2%)	1/118 (0.8%)	46/300 (15.3%)
Clinical symptoms	Motor symptoms, overall	150/418 (35.9%)	65/118 (55.1%)	85/300 (28.3%)
	Dysarthria	67/418 (16.0%)	27/118 (22.9%)	40/300 (13.3%)
	Aphasia	113/418 (27.0%)	31/118 (26.3%)	82/300 (27.3%)
	Sensory symptoms, overall	107/418 (25.6%)	33/118 (28.0%)	74/300 (24.7%)
	Dizziness	81/418 (19.4%)	11/118 (9.3%)	70/300 (23.3%)
	Accompanying non-specific symptoms	110/418 (26.3%)	10/118 (8.5%)	100/300 (33.3%)

Values are *n*/*N* (%). DWI-positive lesions were defined by DWI hyperintensity with corresponding ADC reduction. NIHSS-related percentages are based on available NIHSS data (117 DWI-positive, 288 DWI-negative).

In univariate analysis, DWI positivity was more frequent in patients with cardiovascular risk factors than in those without (36.0% vs. 10.9%; *p* < 0.001). Cardiac comorbidity was likewise associated with higher DWI positivity (47.4% vs. 24.0%; *p* < 0.001).

Among clinical-neurological variables, motor symptoms were significantly associated with DWI positivity (43.3% vs. 19.8%; *p* < 0.001). An NIHSS score of 1–5 and acute symptom onset were also associated with higher DWI-positive rates (42.5% vs. 23.2%, *p* < 0.001; 31.2% vs. 18.1%, *p* = 0.013). NIHSS data were available for 405/418 patients (96.9%).

Several variables were associated with a lower probability of DWI-positive lesions. Non-classical visual symptoms without hemianopia showed no DWI-positive lesions (0/27; *p* < 0.001) and were therefore not included in multivariable regression. Additional factors associated with lower DWI positivity included subacute symptom onset (2.1% vs. 31.5%; *p* < 0.001), isolated dizziness (2.4% vs. 31.1%; *p* < 0.001), accompanying non-specific symptoms (9.1% vs. 35.1%; *p* < 0.001), dizziness overall (13.6% vs. 31.8%; *p* = 0.001), and an NIHSS score of 0 (22.7% vs. 43.1%; *p* < 0.001). Key univariate associations are summarized in Table 3.

**Table 3:** Univariate associations with DWI-positive lesions.

Predictor category	Predictor	DWI-positive rate, present vs. absent	Relative risk	*p-*value
Increased probability	Cardiovascular risk factors	36.0% vs. 10.9%	3.32	<0.001
	Motor symptoms, overall	43.3% vs. 19.8%	2.19	<0.001
	Cardiac comorbidity	47.4% vs. 24.0%	1.98	<0.001
	NIHSS score 1–5	42.5% vs. 23.2%	1.84	<0.001
	Acute symptom onset	31.2% vs. 18.1%	1.72	0.013
	Dysarthria	40.3% vs. 25.9%	1.55	0.017
	Age >65 years	33.6% vs. 22.1%	1.53	0.009
Reduced probability	Non-classical visual symptoms without hemianopia	0.0% vs. 30.2%	0.00	<0.001
	Subacute symptom onset	2.1% vs. 31.5%	0.07	<0.001
	Isolated dizziness	2.4% vs. 31.1%	0.08	<0.001
	Accompanying non-specific symptoms	9.1% vs. 35.1%	0.26	<0.001
	Dizziness	13.6% vs. 31.8%	0.43	0.001
	NIHSS score = 0	22.7% vs. 43.1%	0.53	<0.001

Summary of clinically relevant univariate associations with DWI-positive lesions. Relative risks above 1 indicate an increased probability of DWI positivity when the predictor is present. Relative risks below 1 indicate a reduced probability of DWI positivity. *p*-values are based on Chi-square or Fisher’s exact tests, as appropriate.

### 3.2 Multivariable analyses

#### 3.2.1 Multivariable model 1: clinical-neurological model

The first model (*n* = 405; DWI+ = 117) included motor symptoms, NIHSS score 1–5, and acute symptom onset. All three variables remained associated with DWI positivity after adjustment: motor symptoms (*p* < 0.001), NIHSS score 1–5 (*p* = 0.007), and acute onset (*p* = 0.025).

Regarding the potential overlap between motor symptoms and NIHSS score 1–5, no relevant multicollinearity was identified (VIFs 1.000–1.079; tolerance values 0.926–1.000). In sensitivity analyses excluding either motor symptoms or NIHSS 1–5, the direction and statistical significance of the principal associations remained unchanged.

#### 3.2.2 Multivariable model 2: extended clinical and cardiovascular model

The second model (*n* = 418; DWI+ = 118) included motor symptoms, cardiovascular risk factors, cardiac comorbidity, and acute onset. Cardiovascular risk factors showed the strongest association with DWI positivity (*p* < 0.001); motor symptoms and cardiac comorbidity remained significant, whereas acute onset did not (*p* = 0.100). Adjusted odds ratios are shown in Table 4; selected predictors are visualized in Figure 1.

**Table 4:** Multivariable predictors and diagnostic characteristics.

Model	Predictor or criterion	DWI-positive rate, present vs. absent	Adjusted OR (95% CI)	*p*-value	Sens./Spec.	PPV/NPV	DWI-positive, *n*/*N* (%)	Model diagnostics
Clinical-neurological model (*n* = 405)	—	—	—	—	—	—	117/405 (28.9%)	Omnibus *χ*^2^ (3) = 37.395, *p* < 0.001; Nagelkerke *R*^2^ = 0.126
	Motor symptoms, overall	43.3% vs. 19.8%	2.683 (1.685–4.271)	<0.001	0.55/0.72	0.43/0.80		
	NIHSS score 1–5	42.5% vs. 23.2%	1.940 (1.198–3.140)	0.007	0.44/0.76	0.43/0.77	—	
	Acute symptom onset	31.2% vs. 18.1%	1.999 (1.093–3.658)	0.025	0.86/0.26	0.31/0.82	—	
Extended clinical and cardiovascular model (*n* = 418)	—	—	—	—	—	—	118/418 (28.2%)	Omnibus *χ*^2^ (4) = 64.929, *p* < 0.001; Nagelkerke *R*^2^ = 0.207
	Motor symptoms, overall	43.3% vs. 19.8%	2.653 (1.667–4.221)	<0.001	0.55/0.72	0.43/0.80		
	Cardiovascular risk factors	36.0% vs. 10.9%	3.959 (2.110–7.429)	<0.001	0.88/0.38	0.36/0.89	—	
	Cardiac comorbidity	47.4% vs. 24.0%	2.502 (1.443–4.336)	0.001	0.31/0.87	0.47/0.76	—	
	Acute symptom onset	31.2% vs. 18.1%	1.685 (0.905–3.136)	0.100	0.86/0.26	0.31/0.82	—	
Combined clinical criteria	≥2 of 3 clinical-neurological criteria	—	—	—	0.64/0.67	0.44/0.82	—	
Combined clinical and cardiovascular criteria	≥2 of 4 criteria	—	—	—	0.92/0.39	0.37/0.92	—	
	≥3 of 4 criteria	—	—	—	0.58/0.78	0.51/0.83	—	
Low-risk model (*n* = 405)	—	—	—	—	—	—	117/405 (28.9%)	Omnibus *χ*^2^ (4) = 60.743, *p* < 0.001; Nagelkerke *R*^2^ = 0.199
	Subacute symptom onset	2.1% vs. 31.5%	0.075 (0.010–0.568)	0.012	—	—	—	
	Accompanying non-specific symptoms	9.1% vs. 35.1%	0.282 (0.137–0.580)	0.001	—	—	—	
	NIHSS score = 0	22.7% vs. 43.1%	0.443 (0.274–0.714)	0.001	—	—	—	
	Dizziness	13.6% vs. 31.8%	0.473 (0.232–0.967)	0.040	—	—	—	
Combined low-risk criteria	≥2 of 4 criteria	—	—	—	0.11/0.58 (95% CI 0.06–0.18/0.52–0.64)	0.10/0.62 (95% CI 0.05–0.16/0.56–0.68)	13/133 (9.8%; 95% CI 5.3–16.1)	
	≥3 of 4 criteria	—	—	—	0.02/0.84 (95% CI 0–0.06/0.79–0.88)	0.04/0.68 (95% CI 0.01–0.14/0.63–0.73)	2/48 (4.2%; 95% CI 0.5–14.3)	
	4 of 4 criteria	—	—	—	0/0.96 (95% CI 0–0.03/0.93–0.98)	0/0.70 (95% CI 0–0.29/0.66–0.75)	0/11 (0%; 95% CI 0–28.5)	

Displayed sensitivity, specificity, PPV, and NPV are descriptive and were calculated for each clinical criterion or combined threshold. For the combined low-risk criteria, 95% confidence intervals are provided because of the small subgroup sizes; threshold fulfillment was treated as a positive test for DWI positivity. The observation of 0/11 DWI-positive cases among patients fulfilling all four criteria should not be interpreted as reliable exclusion of DWI positivity. NIHSS-based values use available NIHSS data.

**Figure 1:**
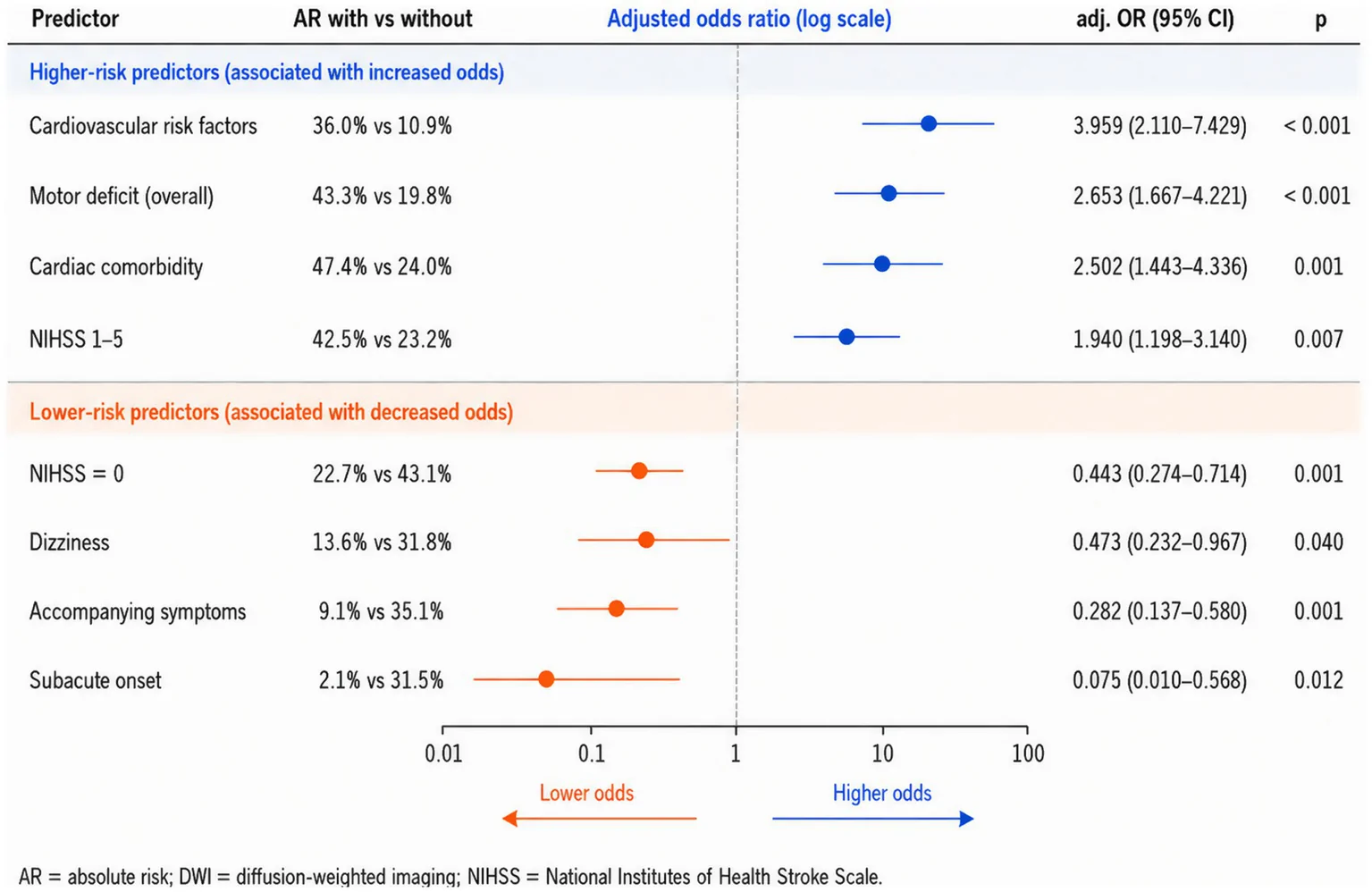
Clinical features associated with DWI-positive lesions. Selected multivariable associations with DWI-positive ischemic lesions. Forest plot of adjusted odds ratios with 95% confidence intervals. The dashed line indicates no association.

#### 3.2.3 Diagnostic characteristics of combined criteria

Sensitivity, specificity, PPV, and NPV varied according to the number of fulfilled criteria in both models. In model 1, fulfillment of ≥2 of 3 criteria yielded a sensitivity of 0.64 and specificity of 0.67. In model 2, a threshold of ≥3 of 4 yielded a sensitivity of 0.58 and specificity of 0.78 (Figure 2, Table 4).

**Figure 2:**
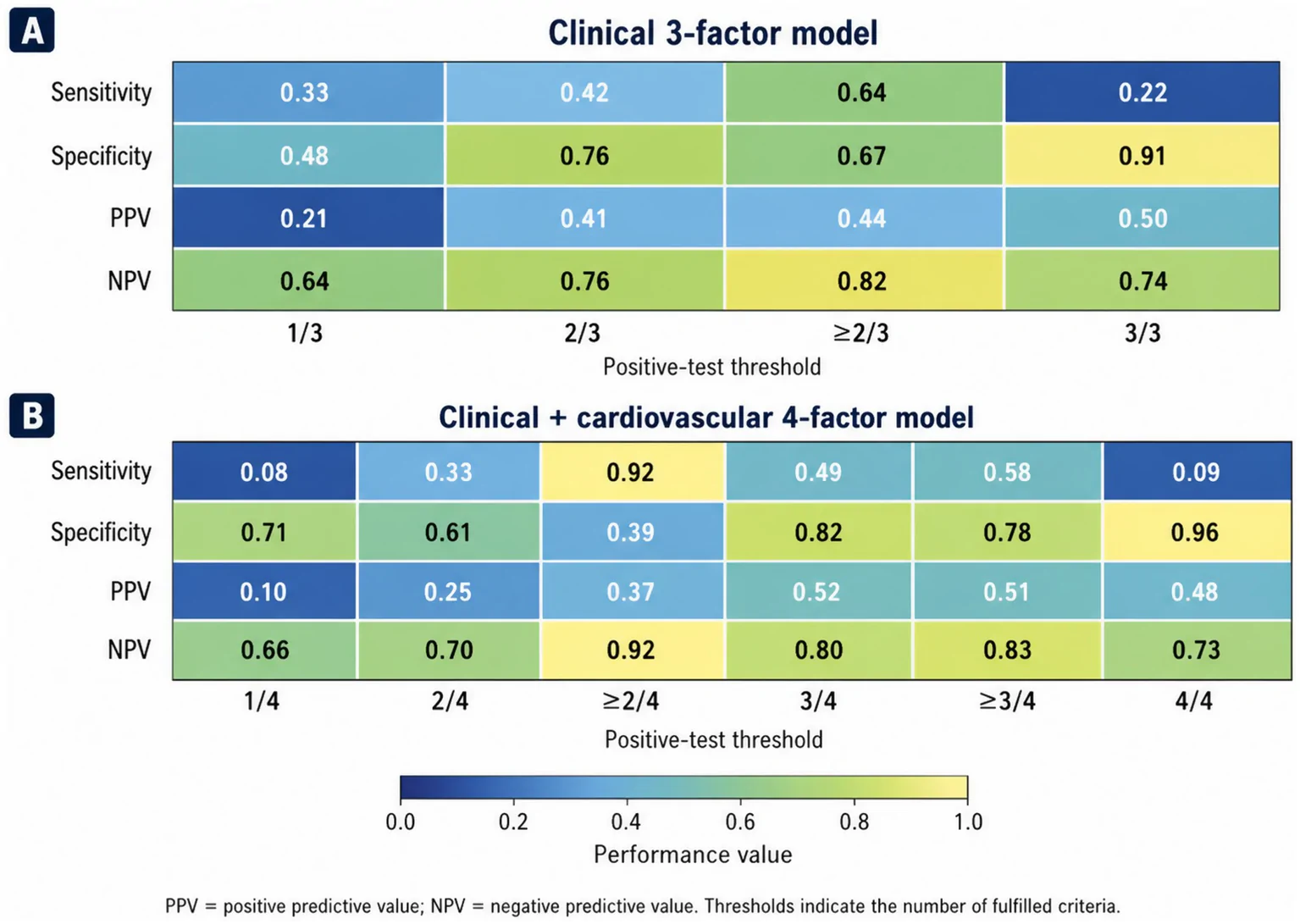
Diagnostic characteristics of combined clinical criteria for DWI-positive lesions. Heatmaps show sensitivity, specificity, PPV, and NPV across thresholds in the clinical 3-factor model **(A)** and clinical-cardiovascular 4-factor model **(B)**. Warmer colors indicate higher values.

#### 3.2.4 Multivariable model 3: low-risk model

The low-risk model (*n* = 405; DWI+ = 117) included subacute onset, accompanying non-specific symptoms, NIHSS score 0, and dizziness as variables associated with lower odds of DWI positivity (Table 4, Figure 1). Subacute onset showed the strongest inverse association (aOR 0.075, *p* = 0.012), followed by accompanying non-specific symptoms (aOR 0.282, *p* = 0.001), NIHSS score 0 (aOR 0.443, *p* = 0.001), and dizziness (aOR 0.473, *p* = 0.040). DWI positivity decreased with an increasing number of low-risk criteria: 13/133 patients with ≥2 criteria (9.8%), 2/48 with ≥3 criteria (4.2%), and 0/11 with all four criteria (Table 4).

In a sensitivity analysis restricted to patients with complete symptom-to-MRI timing and NIHSS data (*n* = 273; 78 DWI-positive events), no statistically significant association between symptom-to-MRI time and DWI positivity was detected after adjustment for the four low-risk features (adjusted OR per 24-h increase, 1.070; 95% CI, 0.610 to 1.875; *p* = 0.814). The study cohort and analysis populations are summarized in Figure 3.

**Figure 3:**
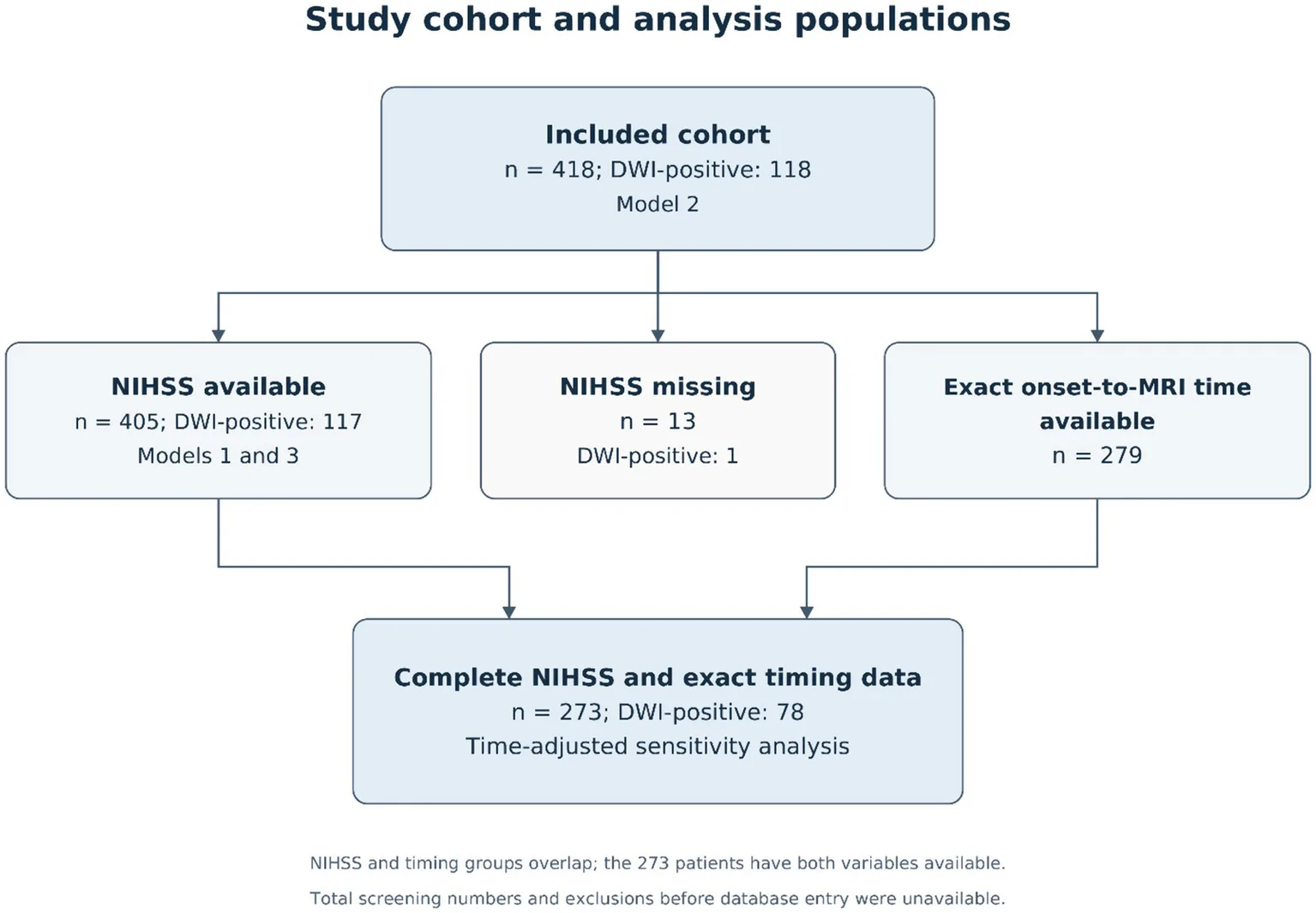
Study cohort, data availability, and analysis of populations. Groups with available NIHSS and symptom-onset-to-MRI data overlap. Screening numbers and exclusions before database entry were not available.

## 4 Discussion

### 4.1 Principal findings

The key finding of this study was that clinically low-risk constellations did not reliably exclude DWI-positive lesions in patients with suspected TIA. Although the likelihood of DWI positivity decreased with an increasing number of low-risk features, DWI-positive lesions were still detected in 9.8% of patients fulfilling ≥2 and 4.2% fulfilling ≥3 low-risk criteria. Thus, a lower probability of DWI positivity should not be equated with reliable exclusion of acute DWI-positive lesions. At the same time, clinical and cardiovascular features stratified the likelihood of DWI positivity and may therefore help estimate expected DWI lesion yield. Whether such information can be used to prioritize MRI requires prospective validation.

### 4.2 Low-risk constellations and DWI lesion yield

The low-risk model identified subacute symptom onset, accompanying non-specific symptoms, NIHSS score 0, and dizziness as variables associated with lower DWI positivity. Importantly, DWI positivity decreased with an increasing number of low-risk criteria, indicating a progressively lower DWI lesion yield. Although no DWI-positive lesion was observed among the 11 patients fulfilling all four criteria, the small size of this subgroup resulted in a wide 95% confidence interval (0.0–28.5%) and precludes conclusions regarding reliable exclusion of DWI-positive lesions.

As a non-specific symptom with a broad differential diagnosis, dizziness was associated with a lower probability of DWI positivity in this cohort. However, dizziness was recorded only as a binary chart-derived variable and was not systematically sub-classified into specific dizziness phenotypes. Therefore, this finding should not be misinterpreted as applying equally to all dizziness presentations. Nor should it be misinterpreted as excluding posterior circulation ischemia, because DWI-positive ischemia may also occur in patients with ataxia, non-focal, or visual symptoms (17, 18). Localizable posterior circulation symptoms therefore require careful diagnostic evaluation. Similarly, non-specific accompanying symptoms were associated with a lower probability of DWI-positive lesions. This may reflect the broad differential diagnosis associated with non-specific symptoms, including TIA mimics.

Our findings illustrate an important distinction between a lower observed probability of DWI positivity and reliable exclusion of DWI-positive lesions. Combinations of low-risk features were associated with progressively lower DWI lesion yield but did not reliably exclude DWI positivity.

### 4.3 Clinical-neurological predictors

DWI-positive lesions were detected in 28.2% of patients in our cohort, with motor symptoms showing the strongest association with DWI positivity among the clinical-neurological features. This supports previous studies reporting motor symptoms as robust clinical markers of ischemia in suspected TIA or minor stroke (18, 19). Similarly, Gallogly et al. (17) reported DWI-positive lesions in 27.4% of patients presenting with suspected TIA or minor stroke. Weakness, dysarthria, and ataxia were associated with DWI positivity, while 38.5% of DWI-positive patients had atypical symptoms.

However, motor symptoms and NIHSS score 1–5 partially overlap conceptually, because motor deficits contribute directly to the NIHSS score. Their simultaneous inclusion should therefore be interpreted as reflecting overlapping measures of neurological deficit burden rather than fully independent effects. Importantly, sensitivity analyses excluding either motor symptoms or NIHSS score 1–5 yielded consistent associations for the remaining neurological feature and acute symptom onset.

In contrast, aphasia was not associated with a higher rate of DWI-positive lesions in our cohort. This finding should be interpreted cautiously, as aphasic symptoms in transient or mild presentations may be difficult to classify retrospectively and may reflect heterogeneous underlying conditions, including stroke mimics. Previous studies have shown that aphasia assessment in the acute setting may be challenging and that isolated aphasia is not invariably attributable to ischemic stroke. In addition, the present study did not differentiate aphasia subtypes or formally assess the diagnostic accuracy of aphasia screening. Therefore, the absence of an association in this cohort should not be interpreted as evidence against the clinical relevance of aphasia in suspected cerebrovascular events (20, 21).

NIHSS score 1–5 was associated with higher DWI positivity, while NIHSS score 0 was associated with lower DWI positivity without excluding it. This may be explained by subtle deficits, posterior circulation symptoms, and transient symptoms that are insufficiently captured by the NIHSS. This finding is clinically relevant because a normal NIHSS cannot be used to rule out DWI-positive minor stroke.

### 4.4 Cardiovascular risk profile

The extended clinical and cardiovascular model showed that the composite variable cardiovascular risk factors had the strongest association with DWI positivity. The variable cardiac comorbidity was also associated with DWI-positive lesions, supporting the relevance of vascular and cardiac profiles in patients with suspected TIA or minor stroke. However, because these composite variables comprised clinically heterogeneous components, the observed associations should not be interpreted as indicating equivalent effects of the individual risk factors or cardiac conditions.

Acute symptom onset was associated with DWI positivity in the clinical-neurological model but lost statistical significance after inclusion of cardiovascular variables. This may reflect overlap between acute onset and vascular risk profile. Clinically, acute onset remains relevant, but it should be interpreted in combination with focal symptoms and vascular risk factors rather than in isolation.

### 4.5 Clinical and imaging implications

Our findings suggest that while clinical and cardiovascular features may help stratify the likelihood of DWI-positive ischemia, even combinations of low-risk features do not reliably exclude acute DWI-positive lesions. This interpretation is consistent with current recommendations emphasizing rapid neurological and imaging-based evaluation in patients with suspected TIA or minor stroke, while recognizing that real-world triage depends on local resources (11, 12). Established clinical risk scores such as the ABCD2 score are also limited when used alone to distinguish high-risk from low-risk patients (22). Our findings further support the observations from the DOUBT study and its *post hoc* analyses by Marko et al., showing that clinical models provide supportive information but have only moderate discriminatory performance (23, 24).

The combined criteria in our study illustrate how clinical stratification may describe differences in expected DWI lesion yield: lower thresholds were associated with higher sensitivity and NPV, whereas stricter thresholds were associated with higher specificity and PPV. Clinical criteria may therefore help estimate the expected DWI lesion yield, but cannot replace DWI-based assessment.

Prospective multicenter studies are needed to determine whether combinations of clinically low-risk features can reproducibly identify patients with very low DWI lesion yield and inform MRI prioritization across different clinical settings, particularly when MRI availability is limited.

### 4.6 Limitations

Several limitations must be acknowledged. First, this was a retrospective single-center study and is therefore susceptible to documentation and selection bias. Second, symptoms were assessed dichotomously and not weighted by severity or duration. Third, the heterogeneous symptom-to-MRI interval represents an important limitation, because DWI visibility changes over time (18). Accordingly, the present analyses describe associations within the clinical cohort studied and should not be interpreted as estimates of DWI detection probability at a defined time point after symptom onset. In addition, symptom onset to MRI timing was not available for all patients, which may have introduced additional bias and limits the representativeness of the timing analysis. Fourth, cardiovascular risk factors and cardiac comorbidities were analyzed as composite variables, which do not account for the potentially different or cumulative contributions of individual risk. Individual component frequencies could not be reliably reconstructed from the analytical dataset. Fifth, motor deficits contribute directly to the NIHSS score, which may introduce collinearity in models including both variables. Additionally, defining the study endpoint as DWI-positive lesions with an ADC correlate does not capture DWI-negative ischemic strokes (9, 10). Finally, MRI was performed on both 1.5 T and 3 T systems, and field strength was not incorporated into the analyses. Although both scanners used the standardized institutional TIA protocol including biplanar DWI acquisition and comparative data have not shown diagnostic superiority of 3 T over 1.5 T DWI in acute stroke, differences in lesion conspicuity, particularly for very small lesions, cannot be excluded (25). Additionally, DWI status was based on routine clinical neuroradiology reports rather than blinded, independent study-specific image reassessment. Complete symptom resolution at presentation was not recorded separately, preventing a reliable subgroup analysis restricted to patients with fully resolved symptoms. The absence of a screening log limits assessment of selection bias related to the institutional MRI pathway. Low-risk feature selection and threshold evaluation were performed *post hoc* in the same cohort without internal or external validation, creating a risk of overfitting and optimistic in-sample estimates.

## 5 Conclusion

Clinically low-risk features were associated with lower DWI lesion yield but did not reliably exclude DWI-positive lesions in patients with suspected TIA. Clinical and cardiovascular features may help estimate the likelihood of DWI positivity; their potential role in MRI prioritization requires prospective validation.

## Data Availability

The raw data supporting the conclusions of this article will be made available by the authors, without undue reservation.
